# Estimation of Critical Gap Based on Raff's Definition

**DOI:** 10.1155/2014/236072

**Published:** 2014-11-09

**Authors:** Rui-jun Guo, Xiao-jing Wang, Wan-xiang Wang

**Affiliations:** ^1^National ITS Center, Research Institute of Highway, Beijing 100088, China; ^2^School of Traffic and Transportation, Dalian Jiaotong University, Dalian 116028, China

## Abstract

Critical gap is an important parameter used to calculate the capacity and delay of minor road in gap acceptance theory of unsignalized intersections. At an unsignalized intersection with two one-way traffic flows, it is assumed that two events are independent between vehicles' arrival of major stream and vehicles' arrival of minor stream. The headways of major stream follow M3 distribution. Based on Raff's definition of critical gap, two calculation models are derived, which are named M3 definition model and revised Raff's model. Both models use total rejected coefficient. Different calculation models are compared by simulation and new models are found to be valid. The conclusion reveals that M3 definition model is simple and valid. Revised Raff's model strictly obeys the definition of Raff's critical gap and its application field is more extensive than Raff's model. It can get a more accurate result than the former Raff's model. The M3 definition model and revised Raff's model can derive accordant result.

## 1. Introduction

### 1.1. Literature Review of Critical Gap

At unsignalized intersections with two traffic flows, vehicles streaming at major road have the priority to pass intersections; however, vehicles streaming at minor road must wait for the enough gap of major stream. Critical gap is the minimum major-stream headway during which a typical minor-stream vehicle can make a maneuver, as discussed by Luttinen [1]. It is an important parameter to determine the capacity and delay of minor road. Critical gap is a judgment threshold to whether a minor-stream vehicle can enter major stream. That is to say, the vehicle can enter intersection when the headway of major stream is larger than critical gap and the headway is called accepted gap, whereas the vehicle cannot enter intersection when the headway is smaller than critical gap and the headway is called rejected gap.

It is obvious that different drivers or the same driver at different times have different critical gaps because of different driving operation. This kind of difference is called inconsistent and nonhomogeneous as discussed by Plank and Catchpole [2]. So critical gap is a random variable and assumed following some distribution which can be described by the average and variance.

Many different methods for estimation of critical gap at unsignalized intersections have been presented. Polus et al. [3] found that critical gap decreased with increase of the waiting time, and the relation between them was “S” type curve which could be expressed by the exponential model. Hamed et al. [4] thought that the distribution of critical gap was related to driving years, social and economic background of drivers, waiting time, and travel destination. The average value of critical gap is related to conflicted flow, the lane number of minor road, proportion of turn-left lane, and the velocity of major stream. They built multiple regression model of critical gap. Ashworth [5, 6] analyzed the distribution character of accepted gap with different flow rate of major road and modeled the average value and variance of critical gap on the assumption that the headways of major road follow negative exponential distribution and critical gap and accepted gap follow normal distribution. Based on Ashworth's model, Miller [7] proposed the model of critical gap provided that it follows *γ* distribution. Raff and Hart [8] regarded the cross point of rejected gap number and accepted gap number as critical gap. The method is widely used in many countries.

Brilon et al. [9] pointed that Siegloch's method was valid only in bunched flow. A majority of methods are valid in free flow; for example, lag method can be regarded as the theoretical reference value. Logit method is similar to the classical logit models of transportation planning. Probit procedure cannot consider critical gap as normal distribution despite its obvious variance. Hewitt's method [10] has a farther development than Probit procedure and even has corresponding calculation software of GAPTIM and PROBIT. The maximum likelihood method [11] estimates the average value and variance on the assumption of normal distribution of accepted gap, maximum rejected gap, and critical gap. Raff and Hart [8] found the maximum likelihood method and Hewitt's method had an accurate result by analyzing and calculating the field data.

### 1.2. Experiential Value of Critical Gap

Roundabout is a type of classic unsignalized intersection. Many researchers obtained different values of critical gap by observation of various types of roundabouts. HCM (see Highway Capacity Manual 2000 [12]) recommends the value range of critical gap *t*_*c*_ and follow-up headway *t*_*f*_ as in Table 1.

Siegloch (see Wu [13]) thought that critical gap could be evaluated by the following equation:

(1) $$\begin{array}{c} t_{c}=t_{0}+0.5t_{f}, \end{array}$$

where *t*_0_ is minimum accepted gap.

Akcelik [14] obtained parameter values at the field roundabouts, *t*_0_ = 3.73 s, *t*_*f*_ = 2.31 s, and *t*_*c*_ = 4.89 s. He found that the fixed critical gap could not be applicable to all roundabouts; contrarily, it fluctuated at the interval of (2.2, 8.8 s) [15].

Australia model (see Akcelik [15]) used fixed follow-up headway and critical gap, which are, respectively, 2 s and 4 s. The follow-up headway changes in the interval of (1.2, 4 s) usually are treated as 2 s when calculating capacity or analyzing operational character at roundabouts. The follow-up headway fluctuates at the interval of (1.8, 3.2 s) in Switzerland (see Lertworawanich and Elefteriadou [16]); nevertheless the usual values are from 1.8 s to 3.0 s.

Tanyel [17] found that the acceptable gap changed at the interval of (4.54, 6.18 s) based on the survey of six roundabouts. The following parameter values (see Tanyel and Yayla [18]) are adopted when minor road vehicles always wait to enter single-lane roundabouts: critical gap is 3.5 s, follow-up headway is 2 s, and minimum headway is 1.8 s.

## 2. Calculation Model of Critical Gap

It is difficult to measure critical gap directly. Usually it can be estimated by accepted gaps and rejected gaps. As mentioned before, there are many calculation methods of critical gap, such as regression method, maximum likelihood method, Siegloch's method, Ashworth's method, Raff's method, Harders' method, Hewitt's method, Logit procedure, and Probit procedure. Ashworth's method and Raff's method are listed as follows.

### 2.1. Raff's Method

The critical lag *L* is the size lag which has the property that the number of accepted lags shorter than *L* is the same as the number of rejected lags longer than *L* (Raff and Hart [8]). A similar definition was proposed by Drew [19] but for gaps rather than lags. So critical gap can be derived from the cross point between the number of curves of accepted gaps and rejected gaps.

Raff's method can be expressed as (2) (see Brilon et al. [9]). Consider

(2) $$\begin{array}{c} 1-F_{r}\left(t\right)=F_{a}\left(t\right), \end{array}$$

where *t* is headway of major stream; *F*_*a*_(*t*) is cumulative probability of accepted gap; *F*_*r*_(*t*) is cumulative probability of rejected gap.

Raff's method is also called threshold method. The flow rate of major road has a prominent influence on critical gap value. The method is used widely in many countries owing to its simplicity and practicality.

### 2.2. Ashworth's Method

Based on the assumption that the headway of major stream follows negative exponential distribution and critical gap and the accepted gap follow normal distribution, Ashworth gave the calculation formula of critical gap as follows:

(3) $$\begin{array}{c} {\overset{-}{t}}_{c}={\overset{-}{t}}_{a}-q\sigma_{a}^{2}, \end{array}$$

where ${\overset{-}{t}}_{c}$ is average critical gap (s), *q* is flow rate of major stream (veh/s), ${\overset{-}{t}}_{a}$ is average accepted gap (s), and *σ*_*a*_^2^ is variance of accepted gaps (s^2^).

Similarly, Miller [7] gave the calculation equation (4) based on the hypothesis that critical gap followed *γ* distribution. Consider

(4) t−c=t−a−Vpσc2,σc=σat−ct−a,

where *σ*_*c*_^2^ is variance of critical gap (s^2^).

The computer iteration can be applied to calculate critical gap using (4). We calculated critical gap by the use of (3).

## 3. Critical Gap Based on Raff's Definition

### 3.1. Assumption

For simplicity, the following special condition is based on the assumption of independence between arrival times of the minor-stream vehicles and the ones of the major-stream vehicles.

Based on the assumption, the distribution form of all headway samples in major stream should be the same as the distribution form of part of stochastic samples when minor-stream vehicles arrive before the intersection. Therefore, we can simulate the headway distribution in the major stream by using the latter samples. These headway samples can be divided into accepted headway and rejected headway, because all surveyed headway samples are related to accepted maneuver or rejected maneuver of the minor-stream vehicles. The distribution of accepted headway and rejected headway can be modeled. Their proportions can be calculated as total accepted coefficient and total rejected coefficient.

The following two circumstances are discussed. (1) In general circumstance, critical gap is a random variable. Based on Raff's definition, the estimation of critical gap is denoted as ${\hat{t}}_{c}$. (2) In special circumstance, critical gap follows normal distribution and can be estimated by the average value ${\overset{-}{t}}_{c}$ and variance *σ* ^2^. The relation between ${\overset{-}{t}}_{c}$ and ${\hat{t}}_{c}$ is discussed.

### 3.2. Definition of Variables

The distribution of headway is very important for the calculation of capacity in gap acceptance theory. Based on the negative exponential distribution of headway (M1) and shifted negative exponential distribution (M2), a bunched exponential distribution (M3) was proposed (Cowan [20]). Generally, an M3 distribution is as follows:

(5) ft=αλe−λt−tmt≥tm0t<tm,Ft=1−αe−λt−tmt≥tm0t<tm,

where *f*(*t*) is probability density function of headway in major stream; *F*(*t*) is cumulative probability function of headway in major stream; *λ* is decay constant (veh/s), *λ* = *αq*/(1 − *qt*_*m*_); *t*_*m*_ is minimum headway in major stream (s); *α* is proportion of free vehicles.

Provided that the headway of major stream follows M3 distribution, headway samples are extracted from major-stream headways when minor-stream vehicles arrive before an intersection. Some variables are defined as follows:

  
*β*_*a*_ is total accepted coefficient or the proportion of accepted gap number *N*_*a*_ to total gap number *N*;
 
*β*_*a*_ = *N*_*a*_/*N*;
 
*β*_*r*_ is total rejected coefficient or the proportion of rejected gap number *N*_*r*_ to total gap number *N*;
 
*β*_*r*_ = *N*_*r*_/*N*; the relation between *β*_*a*_ and *β*_*r*_ is
  (6) $$\begin{array}{c} \beta_{a}+\beta_{r}=1. \end{array}$$

### 3.3. First Circumstance

Raff considered that the number of rejected gaps larger than critical gap was equal to the number of accepted gaps smaller than critical gap. Based on the Raff's definition of critical gap, the equation can be directly expressed as

(7) $$\begin{array}{c} N_{a}F_{a}\left({\hat{t}}_{c}\right)=N_{r}\left[1-F_{r}\left({\hat{t}}_{c}\right)\right]; \end{array}$$

then

(8) $$\begin{array}{c} F_{a}\left({\hat{t}}_{c}\right)=\frac{\beta_{r}}{\beta_{a}}\left[1-F_{r}\left({\hat{t}}_{c}\right)\right]. \end{array}$$

As mentioned earlier, Raff's definition can be expressed as (2). Equation (2) is only the special circumstance of (8) when *β*_*a*_ = *β*_*r*_. So (8) is the real equation which strictly accords with Raff's definition. We name it revised Raff's equation.

Based on Raff's definition of critical gap, the proportion of rejected gaps larger than critical gap is equal to the proportion of accepted gap smaller than critical gap because *N* is fixed. Two proportions can be counteracted, so the total accepted coefficient is equal to the accumulative probability of headway *t* larger than critical gap. The equation can be derived as follows:

(9) βa=PT≥t^c+βaFat^c−βr1−Frt^c=PT≥t^c=∫t^c∞ftdt=1−Ft^c=αe−λt^c−tm;

then

(10) $$\begin{array}{c} {\hat{t}}_{c}=t_{m}-\frac{1}{\lambda }\ln\left(\frac{\beta_{a}}{\alpha }\right), \end{array}$$

where *P*{·} is probability of gap interval.

Equation (10) is based on the M3 distribution of headway in major stream. We name it M3 definition method. Both methods can be seen in the reference (see Guo and Lin [21]).

### 3.4. Second Circumstance and Relation between ${\overset{-}{t}}_{c}$ and ${\hat{t}}_{c}$

Some assumptions are needed. (1) Critical gap follows normal distribution, *t*_*c*_ ~ *Z*(*u*, *σ* ^2^). (2) The headway in major stream follows M3 distribution. (3) The distribution of critical gap is independent of the headway distribution.

For discriminating from the first circumstance, the probability density function of headway in major stream is denoted as *f*_*T*_(*t*), and *f*_*T*_(*t*) = *f*(*t*). The accumulative probability function of headway is denoted as *F*_*T*_(*t*). The probability density function and accumulative probability function of critical gap are denoted as *f*_*TC*_(*t*_*c*_) and *F*_*TC*_(*t*_*c*_) separately.

It has been mentioned as before that

(11) $$\begin{array}{c} \beta_{a}=P\left\{t\geq t_{c}\right\}=P\left\{t-t_{c}\geq 0\right\}. \end{array}$$

Provided that *z* = *t* − *t*_*c*_, its probability density function is *f*_*Z*_(*z*) and accumulative probability function is *F*_*Z*_(*z*). *z* ≥ *t*_*m*_ − *t*_*c*_ and *z* is continuous at 0 for *t* ≥ *t*_*m*_. Consider

(12) βa=Pz≥0=1−Pz<0llll=1−Pz≤0=1−FZ0,βr=FZ0.

*f*_*Z*_(*z*) can be derived by use of *f*_*T*_(*t*) and *f*_*TC*_(*t*_*c*_). According to the assumption of independence, the distribution of *t*_*c*_ can be derived by convolution formula about two independent variables:

(13) fZz=fTtfTCtc=∫−∞+∞fTtc+zfTCtcdtc=∫−∞+∞αλe−λtc+z−tm12πσe−(tc−u)2/2σ2 dtc=αλ2πσ∫−∞+∞e−tc2−2utc+u2+2σ2λtc/2σ2−λz+λtm dtc=αλ2πσeλtm−z−u+σ2λ2/2∫−∞+∞e−(tc−u−σ2λ)2/2σ2 dtc=αλ2πσeλtm−z−u+σ2λ2/22πσ=αλe−λz+u−σ2λ/2−tm.

*z* follows M3 distribution. When *z* = −*u* + (*σ* ^2^ *λ*/2) + *t*_*m*_,

(14) FZz=∫−∞−u+σ2λ/2+tmfZzdz=1−α∫−u+σ2λ/2+tm+∞fZzdz=1−∫−u+σ2λ/2+tm+∞αλe−λz+u−σ2λ/2−tm dz=1+αe−λz+u−σ2λ/2−tm−u+σ2λ/2+tm∞=1−α.

*t* follows M3 distribution, and

(15) $$\begin{array}{c} P\left\{t=t_{m}\right\}=1-\alpha . \end{array}$$

Similarly,

(16) $$\begin{array}{c} P\left\{z=-u+\frac{\sigma^{2}\lambda }{2}+t_{m}\right\}=1-\alpha . \end{array}$$

When *z* ≥ *t*_*m*_ + (*σ* ^2^ *λ*/2) − *u*,

(17) FZz=1−α+∫−u+σ2λ/2+tmzαλe−λz+u−σ2λ/2−tm dz=1−αe−λz+u−σ2λ/2−tm.

So, the accumulative probability function of *z* can be shown as follows:

(18) $$\begin{array}{c} F_{Z}\left(z\right)=\left\{\begin{array}{cc} 1-\alpha e^{-\lambda \left(z+u-\left(\sigma^{2}\lambda /2\right)-t_{m}\right)}, & z\geq t_{m}+\frac{\sigma^{2}\lambda }{2}-u\\ 0, & z<t_{m}+\frac{\sigma^{2}\lambda }{2}-u. \end{array}\right. \end{array}$$

The probability density function of *z* is

(19) $$\begin{array}{c} f_{Z}\left(z\right)=\left\{\begin{array}{cc} \alpha \lambda e^{-\lambda \left(z+u-\left(\sigma^{2}\lambda /2\right)-t_{m}\right)}, & z>t_{m}+\frac{\sigma^{2}\lambda }{2}-u\\ 0, & z<t_{m}+\frac{\sigma^{2}\lambda }{2}-u. \end{array}\right. \end{array}$$

When *z* = 0, *t* = *t*_*c*_. So

(20) $$\begin{array}{c} F_{Z}\left(0\right)=1-\alpha e^{-\lambda \left(u-\left(\sigma^{2}\lambda /2\right)-t_{m}\right)}=\beta_{r},\\ \alpha e^{-\lambda \left(u-\left(\sigma^{2}\lambda /2\right)-t_{m}\right)}=\beta_{a},\\ u=t_{m}+\frac{\sigma^{2}\lambda }{2}-\frac{1}{\lambda }\ln\left(\frac{\beta_{a}}{\alpha }\right). \end{array}$$

The average critical gap can be derived as

(21) $$\begin{array}{c} {\overset{-}{t}}_{c}=u=t_{m}+\frac{\sigma^{2}\lambda }{2}-\frac{1}{\lambda }\ln\left(\frac{\beta_{a}}{\alpha }\right)={\hat{t}}_{c}+\frac{\sigma^{2}\lambda }{2}. \end{array}$$

Namely,

(22) $$\begin{array}{c} {\overset{-}{t}}_{c}={\hat{t}}_{c}+\frac{\sigma^{2}\lambda }{2}. \end{array}$$

It is obvious that ${\overset{-}{t}}_{c}={\hat{t}}_{c}$ when *σ* = 0. The average critical gap is equal to Raff's critical gap, which is similar to the first circumstance. ${\overset{-}{t}}_{c}\neq {\hat{t}}_{c}$ when *σ* ≠ 0; the relation of them can be expressed as (22).

## 4. Simulation of Critical Gap

### 4.1. Generation of Rejected Gaps and Accepted Gaps

The headway which follows M3 distribution is simulated in order to analyze various models of critical gap. On the assumption of independence between arrival times of the minor-stream vehicles and the ones of the major-stream vehicles, the headways of major stream are divided into two sets including rejected gap set and accepted gap set. Critical gap can be calculated by various methods using the same traffic flow.

An example is listed to introduce the generation of headways in major stream which follow M3 distribution. The exponential rejected proportion function is assumed (Guo and Lin [21]). So

(23) $$\begin{array}{c} \frac{\lambda }{r}=\frac{\alpha -\beta_{a}}{\beta_{a}}, \end{array}$$

where *r* is rejected coefficient.

For an unsignalized intersection, *q* = 0.25 veh/s, *α* = 0.6, *t*_*m*_ = 2 s, and *r* = 0.365. Other parameters can be calculated from (4), (6), and (23); *λ* = 0.3, *β*_*a*_ = 0.33, and *β*_*r*_ = 0.67. So the accumulative probability function of headway in major stream is

(24) $$\begin{array}{c} F\left(t\right)=\left\{\begin{array}{cc} 1-0.6e^{-0.3(t-2)}, & t>2\\ 0.4, & t\leq 2. \end{array}\right. \end{array}$$

If *U*1 follows uniform distribution, *U*1 ~ *U*(0,1), *U*1 = 1 − 0.6*e* ^−0.3(*t*−2)^, and the headway *t* can be derived as follows:

(25) $$\begin{array}{c} t=2-\frac{10}{3}\text{l}{\text{n}}^{\left(5/3\right)\cdot \left(1-U1\right)}. \end{array}$$

It is obvious that 1 − *U*1 ~ *U*(0,1); then

(26) $$\begin{array}{c} t=2-\frac{10}{3}\text{l}{\text{n}}^{\left(5/3\right)U1}; \end{array}$$

*t* is the variable of headway in major stream. The headway can be simulated by use of “Rnd()” function in Visual Basic software. Headways smaller than 2 s need to be changed into 2 s which means bunched stream.

The rejected probability of *t* can be calculated by use of exponential rejected proportion function (see Guo and Lin, [21]). The state of rejection or acceptation for *t* can be simulated by comparing the rejected probability and another stochastic value *U*2 from “Rnd()” function. If the rejected probability is larger than *U*2, the headway will be rejected; otherwise, it will be accepted. The rejection state is denoted as 0 and the acceptation state is denoted as 1. A part of simulated accepted gaps and rejected gaps are listed in Table 2.

### 4.2. Calculation of Critical Gap with Different Stochastic Seeds

The above simulation process can be realized by use of VB software. The input parameters include *α* = 0.6, *t*_*f*_ = 3 s, *q* = 0.25 veh/s, and *r* = 0.365. The different stochastic sequences are produced with different stochastic seeds from −1 to −10. One thousand headways are simulated and critical gap can be calculated by various methods in Table 3.

From Table 3, the results of Ashworth's method and Raff's method are adjacent; the results of M3 definition method and revised Raff's method are adjacent and larger than the former calculations. The calculations of M3 definition method and revised Raff's method have smaller fluctuation, and the standard deviations are separately 0.085 s and 0.089 s. Ashworth's method has largest fluctuation with different stochastic seeds, and the standard deviation is 0.503 s. The assumption of Ashworth's method is that accepted gap and critical gap follow normal distribution, whereas it is difficult to satisfy the assumption for the field or simulated data.

The calculation values of Raff's method are smaller than ones of revised Raff's method because the proportion of free vehicles in major stream is 0.25 and the ratio of total rejected coefficient and total accepted coefficient is larger than 1.

### 4.3. Calculation of Critical Gap with Different Flow Rates

**Figure 1:**
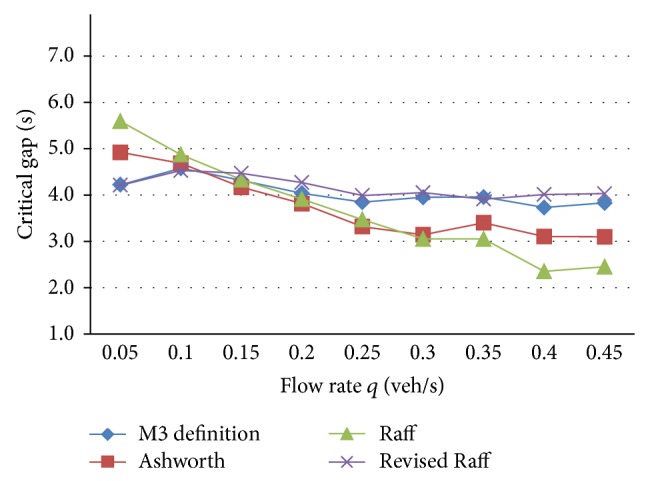
Comparison of critical gap in different traffic rate.

The relation between flow rate and proportion of free vehicles can be treated as *α* = 1 − *qt*_*m*_ (see Wu [13]). When seed = −1, other parameters are similar to the ones in Table 3. Critical gaps with different flow rates are calculated as in Table 4 and the corresponding curve is as in Figure 1.

From Table 4 and Figure 1, critical gaps of all the methods have a trend of decrease with increase of flow rate. The tendency is accordant with field traffic flow. When the major road has a low flow rate, drivers of the minor road are inclined to reject some lager gaps since there are many available large gaps, so critical gap increases. When major road has a high flow rate, drivers are inclined to accept some smaller gaps for the lack of available large gaps in major stream, which is an important reason why drivers will take a risk to enter the intersection with the increase of waiting time, so critical gap decreases.

Ashworth's method and Raff's method have larger fluctuation. M3 definition method and revised Raff's method have smaller fluctuation and their values are adjacent to the recommended range [4.1, 4.6 s] in HCM.

All the methods use the same simulated data; nevertheless, each method has theoretically different calculation value because of their different assumptions. M3 definition method and revised Raff's method are based on the gap acceptance theory. Revised Raff's method can be widely applied. M3 definition method is based on the M3 distribution of major-stream headway. Ashworth's method has a rigorous condition of normal distribution for critical gap and accepted gap which restricts its application scope. So M3 definition method and revised Raff's method are worthy of recommendation.

## 5. Conclusion

Based on gap acceptance theory, two new methods are proposed on the assumption of independence between arrival times of minor-stream vehicles and the ones of major-stream vehicles. New models are verified by simulation of headway data and comparison of various critical gap methods.

Both M3 definition method and revised Raff's method use total rejected coefficient *β*_*r*_. M3 definition method is simple and valid, which can conveniently be substituted into the equations of capacity and delay. Revised Raff's method has more universal application than Raff's method; the calculation value is accurate. Both methods have accordant results, whereas Raff's method and Ashworth's method have larger fluctuation under different circumstance. Ashworth's method needs to satisfy a rigorous assumption condition. M3 definition method and revised Raff's method are worthy of recommendation.

## Figures and Tables

**Table 1:** Critical gap and follow-up headway for roundabouts (HCM 2000 [12]) (s).

	*t*_*c*_	*t*_*f*_
Upper bound	4.1	2.6
Lower bound	4.6	3.1

**Table 2:** Emulated values of rejected gaps and accepted gaps.

Order	*U1 *	Headway (initial)	Headway (*t* = 2, if *t* < *t*_*m*_)	Rejected proportion	*U2 *	State	Rejected gap	Accepted gap
1	0.61	1.96	2.00	1.00	0.64	0	2.00	
2	0.40	3.37	3.37	0.61	0.28	0	3.37	
3	0.89	0.68	2.00	1.00	0.37	0	2.00	
4	0.26	4.85	4.85	0.35	0.67	1		4.85
5	0.50	2.62	2.62	0.80	0.29	0	2.62	
6	0.36	3.70	3.70	0.54	0.50	0	3.70	
7	0.31	4.21	4.21	0.45	0.14	0	4.21	
8	0.75	1.24	2.00	1.00	0.50	0	2.00	
9	0.70	1.48	2.00	1.00	0.63	0	2.00	
10	0.91	0.62	2.00	1.00	0.09	0	2.00	
11	0.99	0.33	2.00	1.00	0.24	0	2.00	
12	0.38	3.51	3.51	0.58	0.82	1		3.51
13	0.98	0.36	2.00	1.00	0.56	0	2.00	
14	0.34	3.91	3.91	0.50	0.04	0	3.91	
15	0.05	10.16	10.16	0.05	0.53	1		10.16

**Table 3:** Critical gap comparison of various methods with different seeds (s) (*t*_*m*_ = 2 s, *α* = 0.6, *t*_*f*_ = 3 s, and *q* = 0.25 veh/s).

Seed	M3 definition	Ashworth	Raff	Revised Raff
	Equation (10)	Equation (3)	Equation (2)	Equation (8)
1	3.90	3.89	3.61	4.07
2	4.00	3.76	3.67	4.13
3	3.86	3.37	3.45	3.85
4	4.06	3.07	3.53	4.09
5	3.99	2.63	3.49	3.91
6	4.12	3.80	3.49	4.05
7	3.99	4.17	3.61	4.05
8	3.95	3.72	3.61	3.97
9	3.98	3.02	3.47	3.99
10	3.84	2.93	3.33	3.93
${\overset{-}{t}}_{c}$	3.97	3.44	3.53	4.00
*σ* ^2^	0.085	0.503	0.101	0.089
Max − min	0.27	1.53	0.34	0.28

**Table 4:** Critical gap comparison of various methods with different flow rates (*t*_*m*_ = 2 s, *t*_*f*_ = 3 s, *r* = 0.365, and seed = −1).

Order	*q* (veh/s)	*α*	M3 definition	Ashworth	Raff	Revised Raff
			Equation (10)	Equation (3)	Equation (2)	Equation (8)
1	0.05	0.9	4.22	4.92	5.59	4.21
2	0.1	0.8	4.58	4.69	4.87	4.53
3	0.15	0.7	4.32	4.17	4.33	4.47
4	0.2	0.6	4.04	3.81	3.91	4.27
5	0.25	0.5	3.85	3.32	3.47	3.99
6	0.3	0.4	3.95	3.14	3.05	4.05
7	0.35	0.3	3.96	3.40	3.05	3.91
8	0.4	0.2	3.73	3.10	2.35	4.01
9	0.45	0.1	3.83	3.10	2.45	4.03

## Notations

### The following symbols are used in this paper.

t: Headway between successive vehicles in major stream
$f\left(t\right)$: Probability density function
$F\left(t\right)$: Cumulative distribution function of headway
α: Proportion of free vehicles
λ: Decay constant (pcu/s)
$t_{m}$: Minimum headway in major stream (s)
$t_{c}$: Critical gap (s)
$t_{0}$: Minimum accepted headway (s)
$t_{f}$: Minimum follow-up headway in minor stream (s)
q: Flow rate of major stream (veh/s)
${\overset{-}{t}}_{c}$: Average critical gap (s)
$\sigma^{2}$: Variance of critical gap (s^2^)
${\overset{-}{t}}_{a}$: Average accepted gap (s)
$\sigma_{a}^{2}$: Variance of accepted gaps (s^2^)
${\hat{t}}_{c}$: Estimation of critical gap in accord with Raff’s definition
$\beta_{a}$: Total accepted coefficient, the proportion of accepted gap number to total gap number
$\beta_{r}$: Total rejected coefficient, the proportion of rejected gap number to total gap number
$F_{a}\left(t\right)$: Cumulative distribution function of accepted gap
$F_{r}\left(t\right)$: Cumulative distribution function of rejected gap
r: Rejected coefficient.
